# Therapeutic potential of recombinant cystatin from *Schistosoma japonicum* in TNBS-induced experimental colitis of mice

**DOI:** 10.1186/s13071-015-1288-1

**Published:** 2016-01-04

**Authors:** Shushu Wang, Yuanyuan Xie, Xiaodi Yang, Xuesong Wang, Ke Yan, Zhengrong Zhong, Xiaowei Wang, Yuanhong Xu, Yi Zhang, Fang Liu, Jilong Shen

**Affiliations:** Department of Immunology, Anhui Medical University, Hefei, 230022 China; Department of Pathogen Biology, Provincial Laboratories of Pathogen Biology and Zoonoses Anhui, Hefei, 230022 China; Pediatrics Department of Affiliated Provincial Hospital, Anhui Medical University, Hefei, 230001 China; Department of Microbiology and Parasitology, Bengbu Medical College; Anhui Key Laboratory of Infection and Immunity, Bengbu, 233000 Anhui China; Department of Laboratory Diagnosis, the Affiliated Hospital of Bengbu Medical College, Bengbu, 233004 China; Department of Laboratory Diagnosis, the First Affiliated Hospital of Anhui Medical University, Hefei, 230022 China

**Keywords:** Cystatin, *Schistosoma japonicum*, Colitis, Immunoregulation

## Abstract

**Background:**

Helminth infections and their components have been shown to have a protective effect on autoimmune diseases. The isolated purified protein from *Schisotosoma japonicum* and its potential therapeutic effect on trinitrobenzene sulfonic acid (TNBS)-induced colitis could provide an alternative way to treat inflammatory bowel disease (IBDs).

**Methods:**

Colitis was induced in Balb/c mice by rectal administration of 2.5 % TNBS, followed by intraperitoneal injection of rSjcystatin 50 μg at 6 h and 24 h afterwards. The inflammation was monitored by recording weight change, stool character and bleeding, colon length, macroscopic score (MAO), microscopic score (MIO), myeloperoxidase activity (MPO) and disease activity index (DAI). The potential underlying mechanism was investigated by examining cytokine profiles including Th1 (IFNγ), Th2 (IL-4), Th17 (IL-17A) and Treg subsets from lymphocytes of spleen, mesenteric lymph nodes (MLN) and intestinal lamina propria mononuclear cells (LPMCs) by flow cytometry. The mRNA relative expressions of the cytokines in splenocytes and MLN were analysed by quantitative real time reverse-transcriptase polymerase chain reaction (qRT-PCR). Simultaneously, the concentrations of the cytokines in the colon homogenate supernatants were tested by enzyme-linked immunosorbent assay (ELISA) and key transcription factors were detected by Western blotting.

**Results:**

Administration of rSjcystatin significantly reduced inflammatory parameters and ameliorated the severity of the TNBS-induced colitis through decreasing IFNγ in three organs and lifting the level of IL-4, IL-13, IL-10, and TGF-β in the colon tissues, with uptrending Tregs in the MLN and LPMC.

**Conclusion:**

The findings provide evidence that rSjcystatin has a therapeutic potential for diminishing colitis inflammation in Balb/c mice. The immunological mechanism may involve the down-regulation of Th1 response and up-regulation of Th2 and Tregs in the MLN and colon.

## Background

Inflammatory bowel disease is characterized by chronic and recurrent inflammatory conditions of the intestinal mucosa, containing two forms, ulcerative colitis and Crohn’s Disease. The pathogenesis of IBD, however, remains unclarified. Recent understanding of the IBD etiology includes combined factors of an individual’s genetics, environmental factors, immune dysregulation, barrier dysfunction and disorder in microbial flora in intestine [1–3], that trigger aggressive cellular immune responses by innate cells, such as dendritic cells and macrophages, leading to production of nitric oxide (NO) and tumor necrosis factor alpha (TNF-α). Intense intestinal inflammation was then initiated by the adaptive immune system that followed [4–6], resulting in stronger production of inflammatory factors such as IFNγ and IL-17A by T helper cells [1].

The incidence of IBDs has increased gradually, about one in every 250 people in some regions of developed countries [7] due to eradication of intestinal helminths and improved domestic hygiene, and is inclined to spread to less-developed countries [8, 9]. In line with the theory of hygiene hypothesis, pre-infections with helminths or treatment with immunomodulatory helminth-derived proteins could suppress inflammation of colitis, which is supported by several studies [10–12]. Infections with *Heligmosomoides polygyrus* have preventative effects on TNBS-induced colitis [13, 14]. Infections with *Trichinella spiralis* [15] or treatment with its larval antigens [16] also display a protective role in dinitrobenzene sulfonate (DNBS)-induced colitis. Besides, the prophylaxis effect of eggs or soluble proteins from *Schistosoma mansoni* on colitis in mice has been found to be associated with down-regulation of the mRNA relative expression of the pro-inflammatory cytokines such as IFNγ and IL-17A, and up-regulation of the anti-inflammatory cytokine IL-4 in the colon [17].

Cystatin, an inhibitor of cysteine protease, comprises a family of cysteine protease inhibitors in various helminthes [18]. It has been reported as an important pathogenicity factor and plays a significant role in suppressing the immune response by regulating inflammatory cytokines [19–23]. Cystatin, which belongs to a large super family of binding inhibitors that interact with papain-like cysteine proteases, has caused much concern of interest with some exciting results, such as its involvement in parasite protease regulation during the molting process of *Onchocerca volvulus* [24, 25], and its modulation of host immune response or inhibition of host cathepsins [26, 27]. Up to now, only two schistosome cystatins have been trialed at the protein level from *S. mansoni* and *S. japonicum* [28, 29]. The full length of genomic DNA sequence of cystatin from *S. japonicum* was available with accession number FJ617451 in GenBank database and the full-length cDNA sequence of Sjcystatin with accession number FJ617450 in GenBank database which was inferred to possess an opening reading frame (ORF) of 306 bp encoding 101 amino acids, with a predicted molecular weight of 11.3 kDa, belonging to family I (stefins) [29]. The recombinant Sjcystatin has been purified and tested to have the inhibitory activity against the cysteine protease papain [29]. Type II family cystatin from *S. japonicum* was recently reported to have a regulatory effect on macrophage skewing in vitro by inhibiting LPS-induced TNF-α and IL-6 production in activated RAW264.7 [30].*.* The in vivo activity of *S. japonicum* cystatin has not been examined so far although it is involved in the in vitro modulation of host immune responses.

In the present study, we observed the therapeutic potential of recombinant cystatin of *S. japonicum* (family I) in TNBS-induced colitis in Balb/c mice. Additionally, we investigated the underlying immunological mechanism of the beneficial effect of rSjcystatin, mainly on Th1, Th2, Th17 and Tregs by determining the cytokine profile of T helper cells isolated from the spleens, mesenteric lymph nodes and the colons, so as to provide a promising strategy for IBD treatment without the infeasible helminth infections in clinical practice.

## Methods

### Animals and TNBS-induced colitis

Female Balb/c mice (specific pathogen free, SPF) of 6 to 8 weeks of age, weighing 18-20 g, were purchased from the Animal Center of Anhui Medical University and allowed to adapt for 4 days before the experiment with food and water free to drink and eat under standard conditions. All procedures were in strict accordance with the Chinese National Institute of Health Guide for the Care and Use of Laboratory Animals, and approved by the Animal Care and Use Committee of Anhui Medical University (approval no: AMU26-08061). Efforts were made to minimize the number of animals and their sufferings.

Colitis was generated by intrarectal administration of TNBS solution (sigma, USA). Briefly, mice were fasted for 24 h with free access to drinking water and then were pressed lightly on the abdomen to facilitate intestinal emptying and anesthetized using sodium pentobarbital (50 mg/kg, ip). Next, the mixture (5 % TNBS and absolute ethyl ethanol with ratio 1:1) was administrated intrarectally via a flexible catheter of 3.5 cm length. After TNBS injection, mice were held in a vertical position for 2 min to prevent leakage of the TNBS mixture and then replaced in the cages with free access to food and water.

### Preparation of recombinant cystatin from *S.japonicum*

The pET-28a-Sjcystatin plasmid was transformed into *E.coli* BL 21 for large scale expression of the recombinant protein induced by 1 mM of isopropylthio-β-galactoside (IPTG, sigma, USA) at 37 °C. The fusion expression of rSjcystatin was purified with a Ni–NTA His* Bind Purification Kit (Merck Millipore, USA). Protein concentration was examined using a Bicinchoninic Acid Protein Assay Kit (Beyotime Biotechnology, China).

### Animal preparation

Mice were divided into ①group TNBS-rSjcystatin, treated with 50 μg rSjcystatin by intraperitoneal injection (i.p.) 6 h and 24 h after TNBS administration; ②group rSjcystatin-TNBS, treated with 50 μg rSjcystatin by i.p. three times at 5d intervals before introduction of colitis; ③group TNBS, treated with TNBS followed by PBS intraperitoneal injection of the same volume as rSjcystatin; ④group rSjcystatin-PBS, treated with 50 μg rSjcystatin by i.p. three times at 5d intervals followed by PBS given intrarectally with the same volume of TNBS mixture; ⑤group PBS-rSjcystatin, treated with 50 μg rSjcystatin by i.p. 6 h and 24 h after PBS given intrarectally with the same volume of TNBS mixture; and ⑥control, injected intrarectally with PBS followed by PBS i.p. in parallel to experimental groups. Three days after colitis induction, mice were sacrificed under euthanasia. Inflammation was determined based on 5 parameters: clinical disease activity, shortening of colon length, macroscopic and microscopic inflammation score, and myeloperoxidase activity in the colon tissue. The grades were conveyed by an investigator blinded for the treatment groups.

Lymphocytes were isolated from the spleens, mesenteric lymph nodes and colons. IFNγ, IL-4, IL-17A, and Tregs were tested by FCM, mRNA relative expressions of cytokines by qRT-PCR and concentrations of the cytokines in colon homogenates tested by ELISA as well as key transcription factors detected by Western blotting.

### Clinical scoring of disease

The mice of each group were observed daily and given a clinical disease score (disease activity index, DAI) ranging from 0 to 12 based on the clinical manifestations of weight loss, diarrhea, and bloody stool [31] (Table 1).

**Table 1:** Disease activity index score parameters (DAI)

Weight loss (%)	Stool	Bloody stool	index
0-1 %	normal	none	0
1-5 %	soft and shaped	between	1
5-10 %	loose	slight	2
10-15 %	between	between	3
>15 %	diarrhea	gross bleeding	4

### Macroscopic inflammation scoring

After being sacrificed, the colon was removed and carefully opened longitudinally. The colonic damage was evaluated macroscopically on the following parameters: extent of adhesions; degree of colonic ulcerations; colonic wall thickness; and degree of mucosal oedema. Each parameter was scored between 0 (normal) and 3 (severe) as described previously [32]. The total score was presented from a minimum of 0 to a maximum of 12. The length of colon, for evaluation of the extent of inflammation, was also measured [33].

### Microscopic inflammation scoring

Colonic segments ready for histopathology examination were fixed in 10 % formalin, then embedded into paraffin, carefully sectioned at 5 μm thickness and stained with hematoxylin and eosin. The histological damage scoring was applied to grade the severity of inflammation based on the 2 following parameters: epithelial lesion (0, none damage; 1, some loss of goblet cells; 2, extensive loss of goblet cells; 3, some loss of crypts; 4, extensive loss of crypts); infiltration (0, none infiltration; 1, infiltration around crypt bases; 2, infiltration spreading to muscularis mucosa; 3, extensive infiltration in the muscularis mucosa with abundant oedema; 4, infiltration spreading to submucosa). The total histological grade ranged from a minimum of 0 to a maximum of 8 as stated above [34].

### MPO activity assay

Myeloperoxidase (MPO) activity was measured by using a MPO assay kit (Nanjing Jiancheng Bio-engineering Institute, China) to detect the degree of myeloid cell infiltration in the colon. Briefly, 100 mg colon tissues were weighed and cut, followed by homogenizing in solution containing 0.9 ml of saline, pH 6.0, 0.5 % hexadecyltrimethyl ammonium hydroxide, and then centrifuged at 12,000 rpm (4 °C) for 15 min. The protein concentration of the colon homogenate supernatants was examined using a Bicinchoninic Acid Protein Assay Kit (Beyotime Biotechnology, China). One hundred microliters of the supernatants were collected and went on following manufacture’ s instructions. The MPO activity of the supernatants was determined and expressed as units per gram of total protein (U/g).

### Cell isolation and preparation

Spleens and MLNs were removed aseptically from experimental mice, and then gently ground in the RPMI 1640 medium. The cells were harvested after mashing through 75 μm cell strainer and suspended in RPMI 1640 supplemented with 10 % bovine fetal serum, 2 mM L-glutamine, 100 U/ml penicillin, and 100 mg/ml streptomycin. LPMCs were isolated as follows [13]. Briefly, colons were opened longitudinally, cut into 0.5 cm pieces after washing thoroughly, and then incubated in D-hank’s with 0.5 mM EDTA at 37 °C for 20 min with gentle shaking. After repeating twice, the tissue was incubated for 35 min at 37 °C in 20 ml RPMI 1640 containing 25 mM HEPES buffer, 2 mM L-glutamine, 5 × 10^−5^ M β-mercaptoethanol,1 mM sodium pyruvate, 100 U/ml penicillin, 10 mg/ml gentamycin, 100 mg/ml streptomycin, as well as 1 mg/ml collagenase IV (Sigma, American). The cell suspension was filtered through a 100 μm filter and washed, and the LPMCs were collected by discontinuous 40/70 % percol gradient centrifugation.

### Flow cytometry analysis of lymphocytes

Lymphocytes from spleens, mesenteric lymph nodes and LPMCs were suspended again and adjusted to the proper cell number. The lymphocytes were stimulated with PMA and ionomycin for 4 h and Golgi inhibitor was then added for the last 2 h of the culture. The cells were subjected to FITC-labeled anti-mouse CD4 (BD Pharmingen), followed by PE-labeled anti-mouse IFNγ (BD Pharmingen), APC-labeled anti-mouse IL-4 (BD Pharmingen) and PE-labeled anti-mouse IL-17A (BD Pharmingen) for intracellular cytokine staining after using the Cytofix/Cytoperm kit (BD Pharmingen) according to the manufactures’ instructions.

For Tregs examination, cells were first stained with FITC-labeled anti-mouse CD4 and APC-labeled anti-mouse CD25 (BD Pharmingen). After surface staining, the cells were stained with PE-labeled anti-mouse Foxp3 antibodies after incubation with Fix/Perm Buffer (BD Pharmingen) at 2-8 °C for 40–50 min in protection from light. Cells were analysed on a flow cytometer.

### RNA extraction and qRT-PCR

The total RNA of lymphocytes from the spleen and MLN was extracted and then reversely transcribed to cDNA using Prime Script 1st Strand cDNA Synthesis Kit (Takara, Japan). A quantitative analysis of the relative mRNA expression of different cytokines was performed to determine the balance between Th1, Th17, Th2, and Treg cells in the lymphocytes from spleen and MLN using qRT-PCR. The parameters for PCR amplification were 95 °C for 5 min, followed by 40 cycles of 95 °C for 10 s and 60 °C for 34 s. GAPDH was used as an endogenous housekeeping gene to normalize the results. The relative mRNA expression was calculated with the comparative △Ct method using the formula 2^-△△Ct^. The forward and reverse primers are listed in Table 2.

**Table 2:** The primers of quantitative RT-PCR

Primer	Forward	Reverse
IFN-γ	GGTCAACAACCCACAGGTCC	CGACTCCTTTTCCGCTTCC
IL-4	TCTCGAATGTACCAGGAGCCATATC	AGCACCTTGGAAGCCCTACAGA
IL-10	GCTCCTAGAGCTGCGGACT	TGTTGTCCAGCTGGTCCTTT
IL-17A	AGGACGCGCAAACATGAGTC	TTGGACACGCTGAGCTTTGAG
TGF-β	CTGGATACCAACTACTGCTTCAG	TTGGTTGTAGAGGGCAAGGACCT
Foxp3	ACACCCAGGAAAGACAGCAAC	CTCGAAGACCTTCTCACAACCAG
GAPDH	CAACTTTGGCATTGTGGAAGG	ACACATTGGGGGTAGGAACAC

### Enzyme-linked immunosorbent assay (ELISA)

ELISA was used to detect values of cytokines in the colon homogenate supernatants following the manufactures’ instructions (RayBiotech, USA).

### Western blotting of transcription factors in colon tissues

One hundred micrograms of colon homogenate proteins were boiled with loading buffer and separated by 12 % polyacrylamide gel electrophoresis. Proteins were blotted onto a polyvinylidene fluoride (PVDF) membrane. Anti-T-bet, Anti-GATA-3, Anti-Foxp3, and Anti-RORγ(t) were applied at proper dilution ratio and GAPDH was used at 1:2000.

### Statistical analysis

Data were evaluated and analysed using a one-way ANOVA analysis and SPSS 16.0 software. *P* < 0.05 was considered as statistically significant. All statistical analysis were performed using GraphPad Prism software.

## Results

### Effect of rSjcystatin on alleviation of the severity of TNBS-induced colitis

**Fig. 1:**
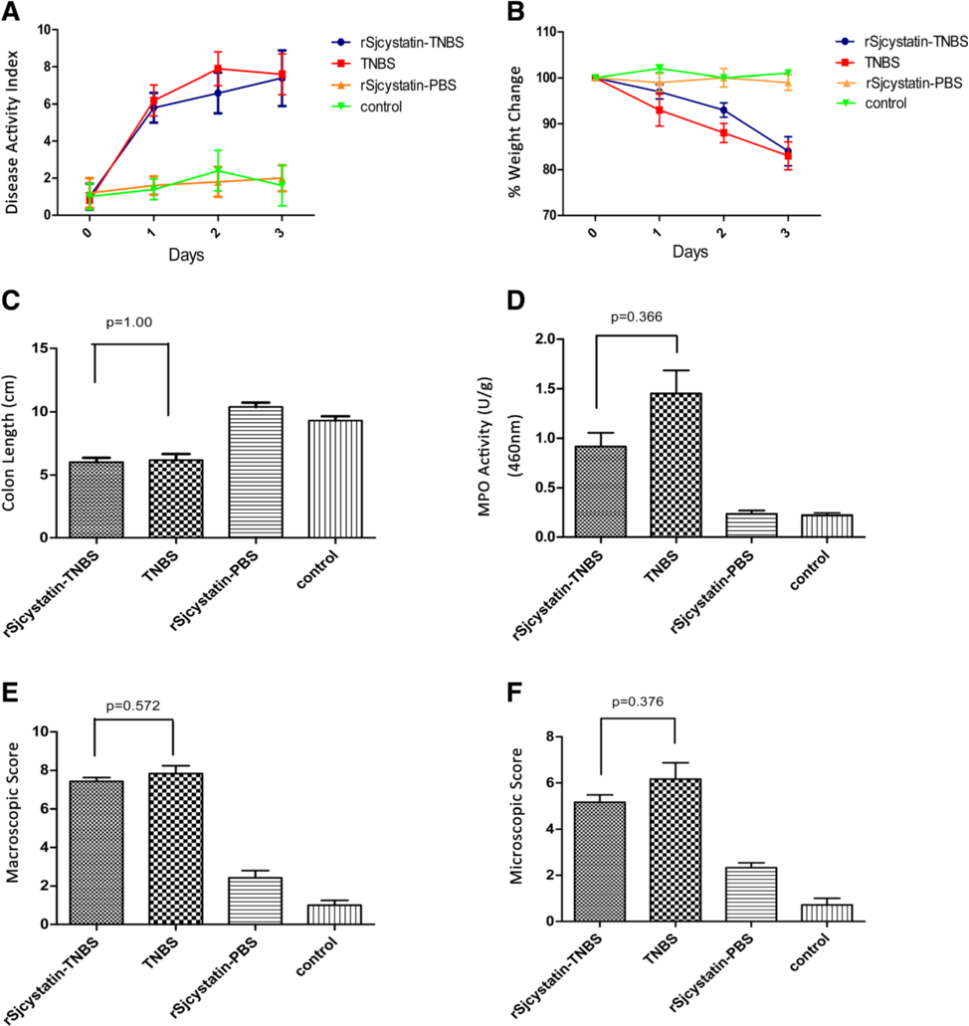
rSjcystatin injection prior to colitis failed to decrease inflammatory index. Disease activity index was evaluated during the experimental period **a** Weight change **b** The colons of four groups were removed and the length was measured and recorded **c**, with MPO activity **d** tested, as well as Macroscopic **e** and Microscopic score **f** No statistical difference of the inflammatory index was displayed between group rSjcystatin-TNBS and group TNBS

**Fig. 2:**
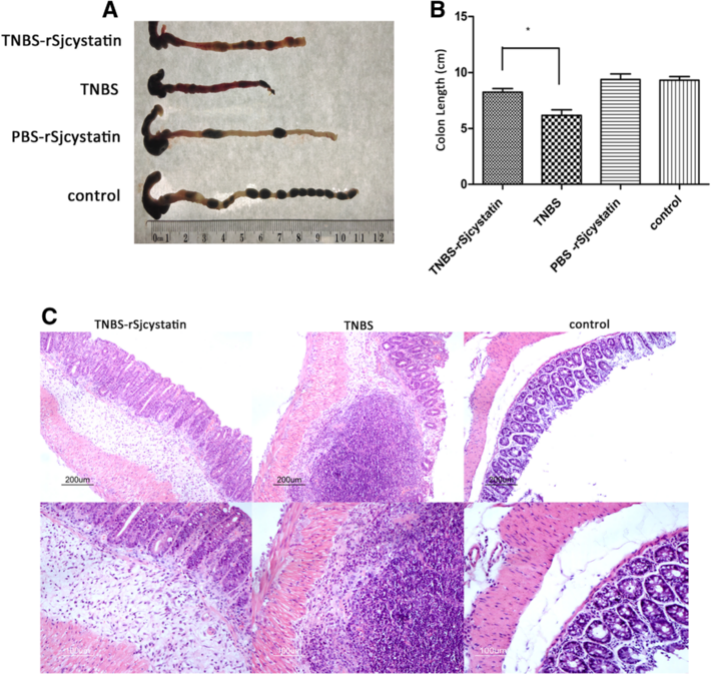
Treatment of rSjcystatin after colitis could diminish colonic inflammation. Colons were removed and shown in (**a**), length was measured with the result of less colon shrinkage in group TNBS-rSjcystatin than group of the untreated colitis mice (**b**), inflammatory cell infiltration in the colon histological sections (**c**)

**Fig. 3:**
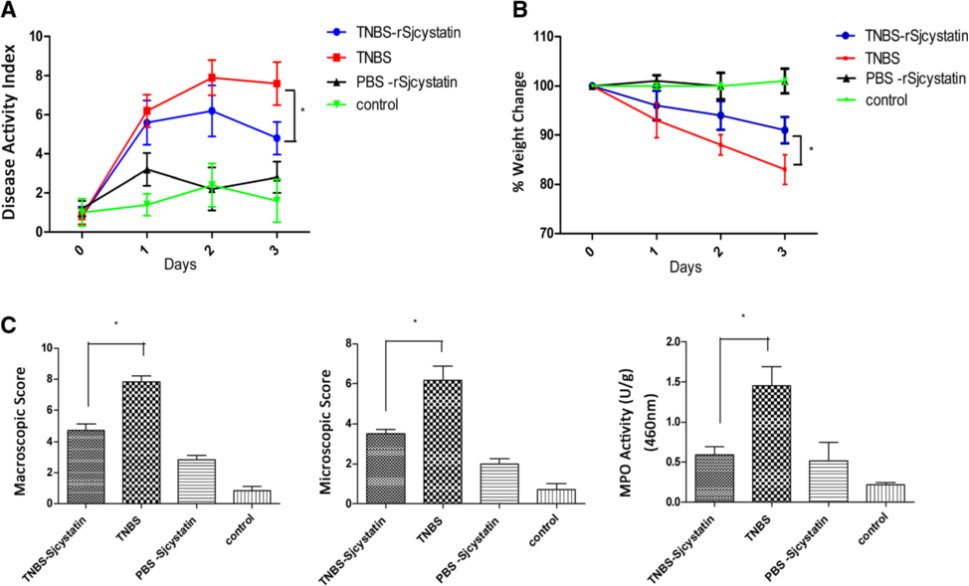
rSjcystatin application lowered the inflammatory index in the mice of TNBS-rSjcystatin group. Disease activity index was evaluated during the experimental period (**a**) Weight loss was monitored daily (**b**) MPO activity, Macroscopic and Microscopic scores in the colon of four groups were compared (**c**) (*p < 0.05). Statistical difference of the inflammatory index was displayed between group TNBS-rSjcystatin and group TNBS

The inflammation was evaluated for weight loss, stool character and expressed as DAI. Three days post-TNBS administration, mice developed significant colitis with manifestations of weight loss, diarrhea, bleeding faeces, and physical weakness compared to the control group treated with PBS only. No macro and microscopic lesions were found in control animals. Weight loss of the experimental mice reached to the maximum of 15 % 3 days post-TNBS administration when DAI increased to (7.60 ± 1.10) vs (8.33 ± 1.97) (*P* > 0.05) in the group of mice with injection of 50 μg rSjcystatin three times prior to colitis. Mice of group rSjcystatin-TNBS developed severe inflammation as TNBS-induced colitis of mice at the level of MPO (0.92 ± 0.28 vs 1.45 ± 0.52) U/g, MAO (7.14 ± 0.69 vs 7.83 ± 0.98) and MIO (5.17 ± 0.75 vs 6.17 ± 1.72) 3 days afterwards (Fig. 1). While injected twice with 50 μg rSjcystatin after colitis, mice showed a significantly lower levels of MPO (0.59 ± 0.23) U/g, MAO (4.71 ± 1.11), MIO (3.50 ± 0.55), DAI (4.80 ± 0.84) and less colon shrinkage than the untreated colitis mice (Figs. 2, 3).

### Measurements of the inflammatory cytokine profiles

**Fig. 4:**
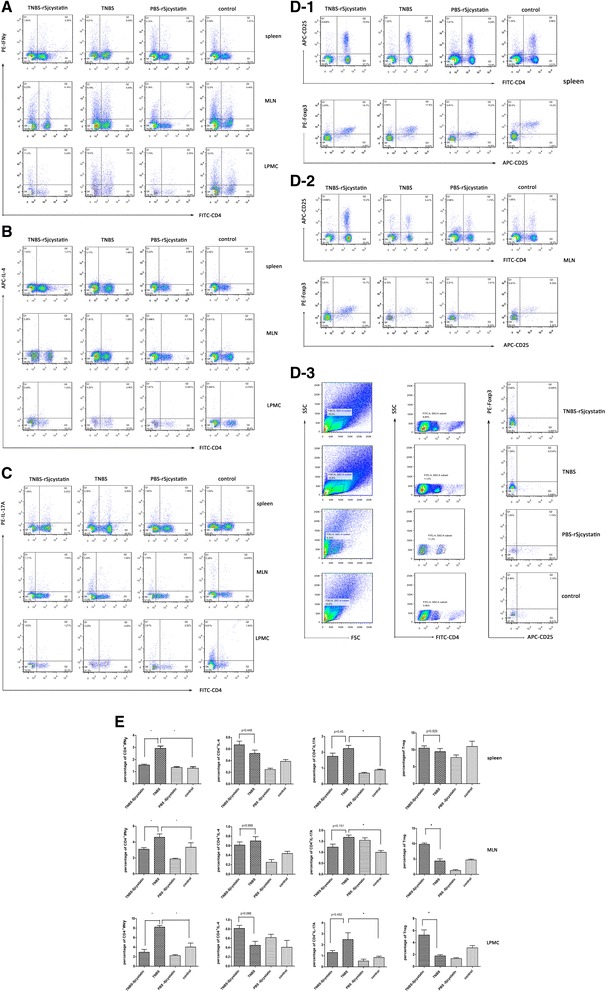
Treatment of rSjcystatin after colitis reduced the percentage of Th1 cell population (CD4^+^IFNγ^+^) in three organs (**a**), increased regulatory T cells (Treg) in the MLN and LPMC (**D**-2,**D**-3). No statistical difference of the percentage of Th2 subsets (CD4^+^IL-4^+^) and Th17 subsets (CD4^+^IL-17A^+^) was noted between colitis group and TNBS-rSjcystatin group (**b,c**). The data were compared (*p < 0.05) (**e**)

**Fig. 5:**
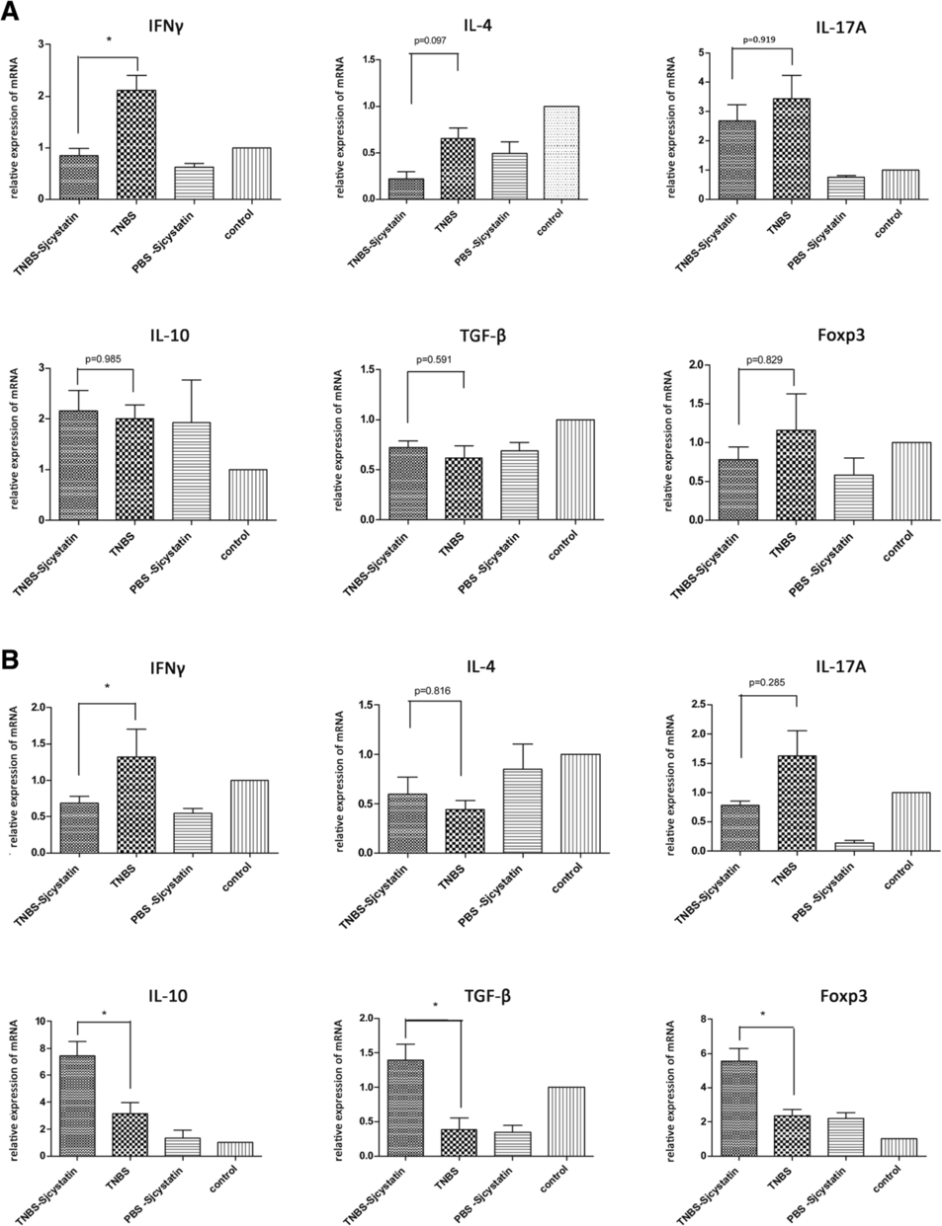
mRNA relative expressions of inflammatory and anti-inflammatory cytokines in the lymphocytes of spleen (**a**) and MLN (**b**) were examined by qRT-PCR (*p < 0.05), showing that IFNγ mRNA expression in the lymphocytes of spleen and MLN increased sharply in the group TNBS, and declined after rSjcystatin application. Nevertheless, IL-10, TGF-β, Foxp3 mRNA relative expressions were lifted in the lymphocytes of MLN but not in the lymphocytes of spleen when rSjcystatin was injected after colitis induction

**Fig. 6:**
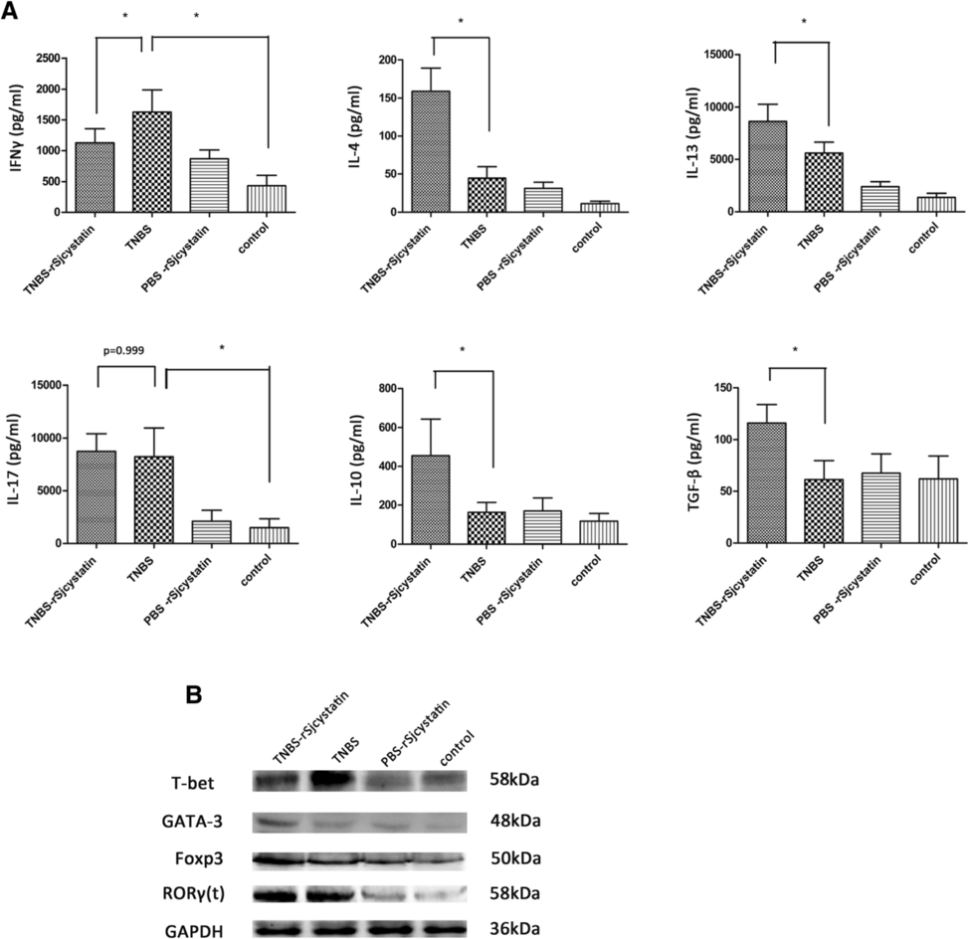
The concentrations of inflammatory and anti-inflammatory cytokines in the colon homogenate supernatants were tested by ELISA (**a**). The level of IFNγ and IL-17 in the colon homogenate supernatants were enhanced in group TNBS, and the level of IFNγ decreased after rSjcystatin injection but IL-17 rejected to decline, while the levels of IL-4, IL-13, IL-10 and TGF-β increased after rSjcystatin injection. Transcription factors of Th1, Th2, Th17, and Treg were detected by Western blotting in the colon tissue (**b**). Higher expressions of GATA-3 and Foxp3 were shown in mice of group TNBS-rSjcystatin than in mice with colitis while a low expression of T-bet was seen after rSjcystatin treatment except that RORγ(t) stayed nearly the same in those two experimental groups

We examined the IFNγ for Th1 response, IL-4 for Th2 response, IL-17A for Th17 and Tregs in the lymphocytes in the spleen, MLN and LPMCs. Compared to the control, Th1 cell populations (CD4^+^IFNγ^+^) and Th17 subsets (CD4^+^IL-17A^+^) increased in the spleen, MLN and colon tissues of mice with colitis (Fig. 4a, c) as well as the enhanced concentrations of IFNγ and IL-17 in colon homogenate supernatants (Fig. 6a). The rSjcystatin application after colitis decreased the proportion of Th1 subsets in these organs, due to the data of the percentage of CD4^+^IFNγ^+^ and relative expression of IFNγ mRNA descended in the spleen and MLN (Fig. 5). Meantime, treatment with rSjcystatin after TNBS induction also led to significant decline of the percentage of CD4^+^IFNγ^+^ in the LPMC (Fig. 4e), which was confirmed in colon homogenate supernatants by ELISA, showing a statistical I difference of IFNγ between mice with colitis (1628.6 ± 358.51) pg/ml and mice treated with TNBS-rSjcystatin (1126.8 ± 230.16) pg/ml (Fig. 6a).

Unexpectedly, the percentages of CD4^+^IL-17A^+^ in the lymphocytes of spleen, MLN and LPMCs exhibited no significant difference in the groups of TNBS and TNBS-rSjcystatin (Fig. 4e). Also, the level of Th17 presented no difference in colon homogenate supernatants of mice in the same groups tested by ELISA and in its transcription factor RORγ(t) detected by protein blotting (Fig. 6).

Moreover, we failed to observe discrepancy in the percentage of CD4^+^IL-4^+^ in the group of TNBS-rSjcystatin and group of TNBS in the three organs (Fig. 4b). But the concentrations of IL-4 (159.95 ± 30.66) pg/ml and IL-13 (8623.1 ± 1644.19) pg/ml in the colon homogenates rose more sharply in the group of TNBS-rSjcystatin when examined by ELISA (Fig. 6a).

As for Tregs, no remarkable difference was noted in the spleen. Tregs number was increased in MLN and LPMC of mice in the group treated with TNBS-rSjcystatin (Fig. 4d). Consistent with the Tregs tested by flow cytometry, relative mRNA expressions of Foxp3, IL-10 and TGF-β in MLNs of mice in the group TNBS-rSjcystatin were markedly enhanced compared with TNBS-treated animals (Fig. 5). In addition, the levels of IL-10 (455.51 ± 187.87) pg/ml and TGF-β (116.18 ± 17.68) pg/ml were elevated higher in the colon homogenate supernatants of mice in the TNBS-rSjcystatin group than in the TNBS group (Fig. 6a).

In order to obtain the most accurate immunological data, we identified the transcription factors of Th1 (T-bet), Th2 (GATA-3), Th17 (RORγ(t)) and Tregs (Foxp3) in colon tissues by Western blotting. GATA-3 and Foxp3 were expressed more highly in mice of group TNBS-rSjcystatin than in mice with colitis, while a low expression of T-bet was seen after rSjcystatin treatment except that RORγ(t) stayed nearly the same in those two experimental groups (Fig. 6b).

## Discussion

Plenty of evidence has demonstrated that helminth and helminth derived products have the ability to suppress the development of IBDs [35–39], mainly through down-regulating Th1 and Th17 responses. The influence of the molecules from *S. japonicum* have not been reported on TNBS-induced colitis so far. Thus the present study aimed to investigate the therapeutic role of rSjcystatin in TNBS-induced colitis in mice. Cystatin, an inhibitor of cysteine protease, was regarded as a key molecule in protein degradation as well as in antigen presentation, apoptosis, protein processing, and inflammation and cancer progression [40–44]. It was reported that cystatin of *filaria* may sharpen T cell proliferation and enhance IL-10 production [20]. Cystatin from *Acanthocheilonema viteae* was noted to be responsible for amelioration of colitis in mice [45].

In the present study, we generated colitis in mice by TNBS, which resembled human inflammatory bowel disease [46] in weight loss, diarrhea, bloody stool, and increased DAI, macro and microscopic score and MPO. Here we found an alleviated inflammation in the colon with low inflammation index when the rSjcystatin was administrated to the mice with colitis at a dose of 50 μg twice after modeling. Unexpectedly, the rSjcystatin, if given to mice before TNBS induction, failed to decrease the levels of DAI, MPO and other inflammation indexes compared to the mice with colitis, suggesting an unascertainable preventative effect of rSjcystatin on TNBS-induced colitis. It is indicated that rSjcystatin acted as an immunoregulator only when the lymphocytes were activated under the inflammatory circumstances.

TNBS-mediated colitis is mainly T cell dependent and CD4^+^T cells are dominantly involved in the pathogenic process [47]. Thus cytokines secreted from CD4^+^T subsets are the value of the targets and will be a potential strategy in IBDs therapy. In the mouse model, we demonstrated that colonic inflammation was caused by the increase of Th1 response along with Th17 activation, which showed the significantly high percentages of CD4^+^IFNγ^+^ and CD4^+^IL17A^+^ in the spleen, MLN and LPMC and the high concentrations of IFNγ and IL-17 in the colon homogenates. It has been widely accepted that Th1 response dominates in the TNBS-induced colitis while IL-17A seems not to be necessary in the T-cell mediated model of colitis [48]. In the present study, the percentage of CD4^+^IFN^+^ decreased in the group TNBS-rSjcystatin whereas CD4^+^IL17A^+^ cells did not show a statistically significant decline, suggesting that rSjcystatin might relieve local inflammation through down-regulating Th1 rather than Th17 response. The result is parallel to the cytokine assay by ELISA and Western blotting in colon tissues. Additionally, Th17 pathobiology is still under controversy for its plastic roles in different experimental models of colitis, distinguished by their function as either “pathogenic” or “nonpathogenic” [49]. It has been reported that IL-17 antibody in the progress of acute colitis amplified the colon inflammation [50] although IL-17A deficiency was found to be able to ameliorate colitis in mice [51].

It is obvious that the Th2 response was initiated in the colon tissue of group TNBSrSjcystatin since high concentrations of IL-4 and IL-13 were noted in colon tissue homogenates, which was verified by its transcription factor GATA-3 protein blotting. Thus a biased Th2 response might be induced by rSjcystatin in the inflammation loci, acting as anti-inflammatory factors down-regulating Th1 response and restraining the colonic inflammation. Similar results have been seen in SEA of *S.mansoni* and in the egg-stage specific antigen from *S. japonicum* that Th2 response was evoked by helminth derived molecules such as omega-1, IPSE/alpha-1 and Cyclophilin A [52–55]. It may be expected that activation of IL-13 [56] and IL-4 signaling might be a potential therapeutic strategy in IBDs. In contrast, IL-4-producing effector T cells in LPMC were not discovered to be at high levels in the mice as expected, which was well in line with the previous report that cytokine detection with ELISA is not necessarily parallel to the intracellular staining analysis by flow cytometry [57].

It has been clarified that regulatory T cells (Tregs) play a crucial role in protection of IBDs in both humans and animals [58, 59]. We discovered that the number of CD4^+^CD25^+^Foxp3^+^ cells stayed stable in the spleens of all experimental groups but dramatically increased in the MLN of the TNBS-rSjcystatin treated mice. Correspondingly, a significant elevation of IL-10, TGF-β and Foxp3 mRNA expressions was found in the MLN of group TNBS-rSjcystatin. Interestingly, numerous unconventional Tregs of CD4^+^CD25^−^Foxp3^+^ were remarkably increased in the LPMCs of colitis mice followed by rSjcystatin injection compared to the mice with colitis. It can be explained by the fact that CD4^+^CD25^−^Foxp3^+^ T cells are the dominant Tregs population in the gut, lung and liver [60] with suppressive activity both in rodents and humans [61, 62]. Similar outcomes of CD4^+^CD25^−^ Foxp3^+^ T cells and their functions were reported in animal models of experimental colitis [63]. It is Foxp3 but not CD25 expression that plays an important role in Tregs functioning of the mouse model [61, 64]. Thus we presumed that this phenomenon might be linked to use of purified protein which took responsibility for the uptrend of CD4^+^CD25^−^Foxp3^+^ population [65]. In accordance with elevated numbers of Th2 and Tregs, IL-10 and TGF-β were simultaneously expressed highly in the homogenates of colon tissues.

We noted that the effect of rSjcystatin was so obvious when given 6 and 24 h after TNBS injection and experimental colitis was significantly dampened 3 days later. Previous researches indicated that macrophages in the intestinal mucosa play a crucial role in the pathogenesis of IBD: low production of inflammatory mediators by macrophage caused by impaired innate immunity results in Crohn’s disease [66, 67]. Thus we presume that rSjcystatin may induce the intestinal macrophages to be more functional as alternatively activated macrophages (M2-like cells) since elevated concentrations of IL-10 and TGF-β in the colon homogenate supernatants were noted. Schnoeller et al. also found that the recombinant cystatin from the filaria *A. viteae* in vivo inhibited acute colitis of mice probably via enhancing IL-10 production by macrophages [45]. Recently, Jang et al. tested recombinant cystatin from *Clonorchis sinensis* in colitis of mice and showed that it could reduce intestinal inflammation related with the recruitment of IL-10 secreting alternatively activated macrophages [68]. The M2-like cells involved in innate immunity may account for the down-regulated impact of rSjcystatin on the proinflammatory cytokine profile in the model colitis although more approaches would need to be done. Additionally, the biological activity of the in vivo effects of rSjcystatin as a protease inhibitor cannot be ruled out due to the fact that rSjcystatin is able to inhibit cysteine protease which is required for the antigen procession and presentation of APCs (antigen presenting cells). So we believe that rSjcystatin could alleviate the impaired host innate immunity soon after being administered to mice with TNBS-induced colitis, followed by initiating the adaptive immunity to stimulate immune regulatory responses. Further investigation would be needed to reveal the precise mechanisms of the inhibitory effect of the helminth-derived peptide on amelioration of inflammatory bowel diseases via innate immunity stimulation.

## Conclusions

Taken together, we demonstrated that the rSjcystatin, derived from *Schistosoma japonicum,* may alleviate the Th1 dominated immunopathogenesis and actively restrain the colonic inflammation in TNBS-induced experimental colitis in mice. The up-regulation of Tregs and Th2 skewed response induced by rSjcystatin in the local inflammatory tissues might be involved in the ameliorated process of colitis.

## Abbreviations

APCs: Antigen presenting cells
DAI: Disease activity index
DNBS: Dinitrobenzene sulfonate
DNBS: Dinitrobenzene sulfonate
ELISA: Enzyme-linked immunosorbent assay
IBD: Inflammatory bowel disease
IPTG: Isopropylthio-β-galactoside
LPMCs: Intestinal lamina propria mononuclear cells
M2: Alternatively activated macrophages
MAO: Macroscopic score
MIO: Microscopic score
MLN: Mesenteric lymph nodes
MPO: Myeloperoxidase activity
NO: Nitric oxide
PVDF: Polyvinylidene fluoride
qRT-PCR: Quantitative real time reverse-transcriptase polymerase chain reaction
SPF: Specific pathogen free
TNBS: Trinitrobenzene sulfonic acid
TNF-α: Tumor necrosis factor alpha
Tregs: Regulatory T cells
